# The complex interplay of antibiotic use and immune checkpoint inhibition

**DOI:** 10.1093/oncolo/oyaf010

**Published:** 2025-02-13

**Authors:** Lawrence W Wu, Ryan H Moy

**Affiliations:** Division of Hematology/Oncology, Department of Medicine, Columbia University Irving Medical Center, New York, NY 10032, United States; Division of Hematology/Oncology, Department of Medicine, Columbia University Irving Medical Center, New York, NY 10032, United States

## The interplay between immune checkpoint inhibition and the gut microbiota

Immune checkpoint inhibitors (ICIs) have fundamentally changed the landscape of treatment for many solid tumors and can lead to durable clinical responses for a significant number of patients with advanced disease.^1^ There have been extensive efforts to identify predictive markers of response and resistance to ICI. One factor of rising interest is the host gut microbiome. Several studies examining the gut microbiota of patients with positive ICI response have identified several bacterial species associated with improved clinical response including *Bifidobacterium*, *Ruminococcaceae*, and *Akkermansia*.^2-4^ These studies examined the stool samples of patients who received ICI treatment and analyzed which bacteria were enriched in patients with ICI response. These bacteria were associated with an increase in effector CD8^+^ and CD4^+^ T cells and dendritic cells, as well as a decrease in regulatory T cells and myeloid-derived suppressor cells. Conversely, *Bacteroides* was identified as a bacterial species enriched in patients who did not respond to ICI.^3^ Importantly, antibiotic exposure can lead to significant changes to the gut microbiota by shifting the composition away from bacteria which promotes more favorable anti-tumor immunity.

## Clinical associations between antibiotic exposure timing and immune checkpoint inhibition

Multiple meta-analyses have identified an overall negative impact of antibiotics on survival outcomes for broad cohorts of patients treated with ICI including those with non-small cell lung cancer (NSCLC), renal cell carcinoma (RCC), melanoma, hepatocellular carcinoma, urothelial cancer, and others.^5,6^ The negative effect appears to be most pronounced when antibiotics are given within 60 days prior to ICI treatment. The antibiotic class of fluoroquinolones has been found to be most strongly associated with worsened clinical response to ICI and may preferentially decrease favorable bacteria species such as *Bifidobacterium* and *Ruminococcaceae*.^7-9^ The context in which antibiotics are used may also influence patient outcomes. A large multi-center retrospective observational study of patients with primarily NSCLC, melanoma, or RCC treated with ICI in Italy identified that antibiotic use in the prophylactic setting for indications such as diverticulitis prophylaxis or to prevent chronic obstructive pulmonary disease was associated with worsened clinical outcomes.^10^ Additionally, exposure to medications such as proton pump inhibitors (PPIs), which may alter the gut microbiota by affecting gastric pH, may additionally impair ICI efficacy.^10-13^ Concomitant use of steroids prior to ICI is associated with unfavorable clinical outcomes, which may be related to alterations in the gut microbiome and inhibition of T cell proliferation and effector T cell cytokine production.^10,14^ Thus, there is a complex interplay of many factors including antibiotic type, timing of antibiotic exposure, and other medications that affect ICI efficacy for patients.

Interestingly, in addition to a negative association with survival outcomes, exposure to antibiotics prior to ICI may also increase risk of immune-related adverse events such as ICI-related colitis in patients with metastatic melanoma and ICI-related pneumonitis in patients with NSCLC.^15,16^ Mechanistically, antibiotic exposure may be related to dysbiosis favoring more pro-inflammatory bacterial species and enhanced activation of pro-inflammatory T cells. Therefore, antibiotic exposure prior to ICI may be associated with multiple negative outcomes for patients.

A recent study published in *The Oncologist* contributes to the growing body of literature exploring the impact of antibiotic exposure and response to ICI. Guerrero et al.^17^ performed a single-center retrospective study in Spain analyzing the associations between antibiotic exposure and outcomes to ICI in a multi-tumor cohort including NSCLC, urothelial cancer, RCC, melanoma, and head/neck cancers. The authors classified the timing of antibiotic exposure relative to ICI treatment into 3 timeframes: “−60 days to +42 days,” “+42 days to +6 months,” and “>6 months.” In the “−60 days to +42 days” subgroup, they identified a lower overall response rate, disease control rate, progression-free survival, and overall survival with ICI treatment. Multivariate analysis accounting for factors such as performance status, tumor type, disease burden, type of ICI, line of treatment, and antibiotic treatment at another time confirmed the survival disadvantage with early antibiotic exposure. Notably, no significant differences were observed when stratifying by antibiotic class and in those who received antibiotics at later time points past 42 days. This study reaffirms findings from prior cohort studies, further emphasizing the potential negative impact of antibiotic exposure on ICI efficacy.

However, context may matter in determining whether antibiotic exposure is necessarily detrimental. In this issue of *The Oncologist*, Zhang et al.^18^ performed a study that challenges the prevailing paradigm suggesting that antibiotics prior to ICI are generally harmful. The authors conducted a single-center retrospective study examining the associations between prior antibiotic use 1–3 months prior to ICI in patients with advanced gastric cancer in China. They further classified antibiotic use into “none,” “prophylactic use,” and “infection.” Notably, 74.8% of patients received antibiotics prophylactically, which was primarily in the context of palliative surgery or abbreviated laparotomy. In this setting, the authors found an improved overall survival benefit with antibiotic use prior to ICI treatment. When further stratified by the use of antibiotics for prophylactic versus active infection, the survival benefit was primarily conferred in the prophylactic antibiotic use cohort. Recognizing the limits of a single-center retrospective study, we believe that this study provides insight into a potential situation in which antibiotic treatment may be associated with benefit in the context of ICI treatment. Further validation in a broader cohort of patients is needed.

There are several outstanding questions that remain:

1. Does the antibiotic class of prophylactic antibiotics affect associations with improved survival benefit in patients with gastric cancer?
2. Are there particular species of bacteria associated with poor response to ICI such as *Bacteroides* that may be preferentially decreased with prophylactic antibiotics?
3. Does concomitant PPI use affect any survival benefit seen with prophylactic antibiotic use?

These clinical findings highlight the multifaceted relationships between ICI treatment response, antibiotic exposure, and the gut microbiota (Table 1). Future studies in this space should stratify by clinical indication for antibiotics, class of antibiotics, timing in relation to ICI, and concomitant medications such as PPIs. We hope that continued research efforts may better define the nuances of these complex interactions and potentially better optimize treatment for patients.

**Table 1.** Factors affecting the relationship between the gut microbiota and immune checkpoint inhibitor response.

	Worse response to ICI	Better response to ICI
Timing of antibiotics	Prior to ICI treatment; highest effect size seen with antibiotics within 60 days prior to ICI	Prophylactic setting in gastric cancer?
Class of antibiotics	Fluoroquinolones may be the highest risk class	Unknown
Enriched stool bacteria species	*Bacteroides*	*Bifidobacterium*, *Ruminococcaceae*, and *Akkermansia*
Concomitant medications	Proton pump inhibitors, steroids	Unknown

## Data Availability

No new data were generated or analysed in support of this research.
